# Supplementary material for: Unveiling the Bioactive Potential of the Invasive Jellyfish Phyllorhiza punctata Through Integrative Transcriptomic and Proteomic Analyses
Source: Biomolecules. 2025 Aug 4;15(8):1121. doi: 10.3390/biom15081121 (PMC12383608; doi:10.3390/biom15081121)
Supplement: Supplementary file 1 [file biomolecules-15-01121-s001.zip › Suplementary Material/FileS3/PEPFOLD4/XP_065070517_12240/clusters.html]

 PEP-FOLD clusters


Download archive of all models  here   

|  |  |  |
| --- | --- | --- |
| # | file | sOPEP |
| # cluster 1 - Size: 8 |  |  |
|  | XP\_065070517\_12240-model1 | -10.8271 |
|  | XP\_065070517\_12240-model1.1 | -10.8271 |
|  | XP\_065070517\_12240-model1.2 | -10.8271 |
|  | XP\_065070517\_12240-model1.3 | -10.8271 |
|  | XP\_065070517\_12240-model1.4 | -10.8271 |
|  | XP\_065070517\_12240-model1.5 | -10.8271 |
|  | XP\_065070517\_12240-model1.6 | -10.6858 |
|  | XP\_065070517\_12240-model1.7 | -10.6858 |
| # cluster 2 - Size: 36 |  |  |
|  | XP\_065070517\_12240-model2 | -10.8137 |
|  | XP\_065070517\_12240-model2.1 | -10.8137 |
|  | XP\_065070517\_12240-model2.2 | -10.8137 |
|  | XP\_065070517\_12240-model2.3 | -10.8137 |
|  | XP\_065070517\_12240-model2.4 | -10.8137 |
|  | XP\_065070517\_12240-model2.5 | -10.8137 |
|  | XP\_065070517\_12240-model2.6 | -10.8137 |
|  | XP\_065070517\_12240-model2.7 | -10.8137 |
|  | XP\_065070517\_12240-model2.8 | -10.8137 |
|  | XP\_065070517\_12240-model2.9 | -10.8137 |
|  | XP\_065070517\_12240-model2.10 | -10.8137 |
|  | XP\_065070517\_12240-model2.11 | -10.8137 |
|  | XP\_065070517\_12240-model2.12 | -10.8137 |
|  | XP\_065070517\_12240-model2.13 | -10.8137 |
|  | XP\_065070517\_12240-model2.14 | -10.8137 |
|  | XP\_065070517\_12240-model2.15 | -10.8137 |
|  | XP\_065070517\_12240-model2.16 | -10.8137 |
|  | XP\_065070517\_12240-model2.17 | -10.8137 |
|  | XP\_065070517\_12240-model2.18 | -10.8137 |
|  | XP\_065070517\_12240-model2.19 | -10.8137 |
|  | XP\_065070517\_12240-model2.20 | -10.8137 |
|  | XP\_065070517\_12240-model2.21 | -10.8137 |
|  | XP\_065070517\_12240-model2.22 | -10.8137 |
|  | XP\_065070517\_12240-model2.23 | -10.8137 |
|  | XP\_065070517\_12240-model2.24 | -10.8137 |
|  | XP\_065070517\_12240-model2.25 | -10.8137 |
|  | XP\_065070517\_12240-model2.26 | -10.8137 |
|  | XP\_065070517\_12240-model2.27 | -10.8137 |
|  | XP\_065070517\_12240-model2.28 | -10.8137 |
|  | XP\_065070517\_12240-model2.29 | -10.8137 |
|  | XP\_065070517\_12240-model2.30 | -10.8137 |
|  | XP\_065070517\_12240-model2.31 | -10.6865 |
|  | XP\_065070517\_12240-model2.32 | -10.6851 |
|  | XP\_065070517\_12240-model2.33 | -10.6851 |
|  | XP\_065070517\_12240-model2.34 | -10.6851 |
|  | XP\_065070517\_12240-model2.35 | -10.6851 |
| # cluster 3 - Size: 56 |  |  |
|  | XP\_065070517\_12240-model3 | -10.7301 |
|  | XP\_065070517\_12240-model3.1 | -10.7301 |
|  | XP\_065070517\_12240-model3.2 | -10.7301 |
|  | XP\_065070517\_12240-model3.3 | -10.7301 |
|  | XP\_065070517\_12240-model3.4 | -10.7301 |
|  | XP\_065070517\_12240-model3.5 | -10.7301 |
|  | XP\_065070517\_12240-model3.6 | -10.7301 |
|  | XP\_065070517\_12240-model3.7 | -10.7301 |
|  | XP\_065070517\_12240-model3.8 | -10.7301 |
|  | XP\_065070517\_12240-model3.9 | -10.7301 |
|  | XP\_065070517\_12240-model3.10 | -10.7301 |
|  | XP\_065070517\_12240-model3.11 | -10.7301 |
|  | XP\_065070517\_12240-model3.12 | -10.7301 |
|  | XP\_065070517\_12240-model3.13 | -10.7301 |
|  | XP\_065070517\_12240-model3.14 | -10.7301 |
|  | XP\_065070517\_12240-model3.15 | -10.7301 |
|  | XP\_065070517\_12240-model3.16 | -10.7301 |
|  | XP\_065070517\_12240-model3.17 | -10.7301 |
|  | XP\_065070517\_12240-model3.18 | -10.7301 |
|  | XP\_065070517\_12240-model3.19 | -10.7301 |
|  | XP\_065070517\_12240-model3.20 | -10.7301 |
|  | XP\_065070517\_12240-model3.21 | -10.7301 |
|  | XP\_065070517\_12240-model3.22 | -10.7301 |
|  | XP\_065070517\_12240-model3.23 | -10.7301 |
|  | XP\_065070517\_12240-model3.24 | -10.7301 |
|  | XP\_065070517\_12240-model3.25 | -10.7301 |
|  | XP\_065070517\_12240-model3.26 | -10.7301 |
|  | XP\_065070517\_12240-model3.27 | -10.7301 |
|  | XP\_065070517\_12240-model3.28 | -10.7301 |
|  | XP\_065070517\_12240-model3.29 | -10.7301 |
|  | XP\_065070517\_12240-model3.30 | -10.7301 |
|  | XP\_065070517\_12240-model3.31 | -10.7301 |
|  | XP\_065070517\_12240-model3.32 | -10.7301 |
|  | XP\_065070517\_12240-model3.33 | -10.7301 |
|  | XP\_065070517\_12240-model3.34 | -10.7301 |
|  | XP\_065070517\_12240-model3.35 | -10.7301 |
|  | XP\_065070517\_12240-model3.36 | -10.7301 |
|  | XP\_065070517\_12240-model3.37 | -10.7301 |
|  | XP\_065070517\_12240-model3.38 | -10.7301 |
|  | XP\_065070517\_12240-model3.39 | -10.7301 |
|  | XP\_065070517\_12240-model3.40 | -10.7301 |
|  | XP\_065070517\_12240-model3.41 | -10.7301 |
|  | XP\_065070517\_12240-model3.42 | -10.7301 |
|  | XP\_065070517\_12240-model3.43 | -10.7301 |
|  | XP\_065070517\_12240-model3.44 | -10.7301 |
|  | XP\_065070517\_12240-model3.45 | -10.7301 |
|  | XP\_065070517\_12240-model3.46 | -10.7301 |
|  | XP\_065070517\_12240-model3.47 | -10.7301 |
|  | XP\_065070517\_12240-model3.48 | -10.7301 |
|  | XP\_065070517\_12240-model3.49 | -10.7301 |
|  | XP\_065070517\_12240-model3.50 | -10.7301 |
|  | XP\_065070517\_12240-model3.51 | -10.7301 |
|  | XP\_065070517\_12240-model3.52 | -10.7301 |
|  | XP\_065070517\_12240-model3.53 | -10.7301 |
|  | XP\_065070517\_12240-model3.54 | -10.7301 |
|  | XP\_065070517\_12240-model3.55 | -10.5772 |
|  |  |  |
